# Cardiorespiratory physiological phenotypic plasticity in developing air‐breathing anabantid fishes (*Betta splendens* and *Trichopodus trichopterus*)

**DOI:** 10.14814/phy2.13359

**Published:** 2017-08-04

**Authors:** Jose F. Mendez‐Sanchez, Warren W. Burggren

**Affiliations:** ^1^ Developmental Integrative Biology Research Group Department of Biological Sciences University of North Texas Denton Texas; ^2^ Department of Biology Autonomous University of the State of Mexico Toluca State of Mexico Mexico

**Keywords:** Circulation, hypoxia, larval fishes, phenotypic plasticity, respiration

## Abstract

Developmental plasticity of cardiorespiratory physiology in response to chronic hypoxia is poorly understood in larval fishes, especially larval air‐breathing fishes, which eventually in their development can at least partially “escape” hypoxia through air breathing. Whether the development air breathing makes these larval fishes less or more developmentally plastic than strictly water breathing larval fishes remains unknown. Consequently, developmental plasticity of cardiorespiratory physiology was determined in two air‐breathing anabantid fishes (*Betta splendens* and *Trichopodus trichopterus*). Larvae of both species experienced an hypoxic exposure that mimicked their natural environmental conditions, namely chronic nocturnal hypoxia (12 h at 17 kPa or 14 kPa), with a daily return to diurnal normoxia. Chronic hypoxic exposures were made from hatching through 35 days postfertilization, and opercular and heart rates measured as development progressed. Opercular and heart rates in normoxia were not affected by chronic nocturnal hypoxic. However, routine oxygen consumption $\dot{M}O_{2}$ (~4 *μ*mol·O_2_/g per hour in normoxia in larval *Betta*) was significantly elevated by chronic nocturnal hypoxia at 17 kPa but not by more severe (14 kPa) nocturnal hypoxia. Routine $\dot{M}O_{2}$ in *Trichopodus* (6–7 *μ*mol·O_2_/g per hour), significantly higher than in *Betta*, was unaffected by either level of chronic hypoxia. *P*_Crit_, the PO_2_ at which $\dot{M}O_{2}$ decreases as ambient PO_2_ falls, was measured at 35 dpf, and decreased with increasing chronic hypoxia in *Betta*, indicating a large, relatively plastic hypoxic tolerance. However, in contrast, *P*_Crit_ in *Trichopodus* increased as rearing conditions grew more hypoxic, suggesting that hypoxic acclimation led to lowered hypoxic resistance. Species‐specific differences in larval physiological developmental plasticity thus emerge between the relatively closely related *Betta* and *Trichopodus*. Hypoxic rearing increased hypoxic tolerance in *Betta*, which inhabits temporary ponds with nocturnal hypoxia. *Trichopodus*, inhabiting more permanent oxygenated bodies of water, showed few responses to hypoxia, reflecting a lower degree of developmental phenotypic plasticity.

## Introduction

Acute hypoxic exposure in aquatic fishes triggers reflex responses aimed at maintaining homeostasis, including reflex branchial hyperventilation – for reviews see (Abdallah et al. 2015; Martin 2014; Milsom 2012; Perry 2011; Porteus et al. 2011). Frequently concurrent with hypoxia‐induced increases in gill ventilation is a reflex bradycardia, and increases in stroke volume and branchial vascular resistance (Farrell 2007; Gamperl and Driedzic 2009; Gamperl and Farrell 2004; Pelster 1999; Stecyk et al. 2008; Tota et al. 2011; Wilson et al. 2015). These physiological and behavioral responses to aquatic hypoxia in fishes – collectively representing the hypoxic ventilatory reflex – are often accompanied by numerous other additional physiological adjustments, including changes in hemoglobin oxygen binding affinity, blood O_2_ carrying capacity, stroke volume, and branchial vascular resistance. All these adjustments to hypoxia can contribute to enhanced O_2_ transfer and potentially lowered ventilatory convection requirement (Gamperl and Driedzic 2009; Perry et al. 2009). The branchial hyperventilation reflex of aquatic fishes minimizes reductions in arterial‐blood PO_2_ associated with aquatic hypoxia, but may also be metabolically expensive (Perry 2011; Perry et al. 2009). Indeed, as aquatic PO_2_ falls, the high cost of gill ventilation with water may become prohibitive, especially when combined with failure of adequate tissue oxygen transport associated with low arterial PO_2_ (Diaz and Breitburg 2009; Farrell and Richards 2009; Graham 1997; Randall et al. 1981).

Although air breathing as a response to aquatic hypoxia is an evolutionarily exotic solution to maintaining ventilation in hypoxic aquatic environments, air breathing has nonetheless independently evolved nearly 50 times in Teleost fishes (Graham 1997; Little 2009; Randall et al. 1981). Air breathing may be a response to nocturnal or seasonal hypoxia in so‐called “facultative” air‐breathing fishes, or may be required in “obligatory” air breathers – for reviews, see (Abdallah et al. 2015; Burggren 1982; Burggren and Johansen 1986; Johansen and Lenfant 1968; Martin 2014; Milsom 2012; Perry 2011; Porteus et al. 2011). The physiological and metabolic responses to hypoxia of adult air‐breathing fishes have been investigated in numerous species. However, natural selection acts very heavily on the embryos, larvae, and juveniles of aquatic and air‐breathing fishes alike, with high mortalities occurring in the earliest developmental stages (Browman 1989; Holzman et al. 2015; Mendez‐Sanchez and Burggren 2014). Thus, understanding the biology of these early stages has additional significance.

In air‐breathing fishes, an initial period of aquatic respiration using a combination of gills and an air‐breathing organ occurs prior to the functional development of both ventilated and perfused air‐breathing organ and the associated onset of air breathing. Yet, relatively few studies have investigated the morphological and physiological transitions to air breathing in larval air breathing fishes (Ahmad and Hasnain 2005; Blank and Burggren 2014; Brauner and Rombough 2012; Burggren 1979; Islam 2005; Liem 1981; Mendez‐Sanchez and Burggren 2014; Terjesen et al. 2001) or, indeed, any tropical species (Peck and Moyano 2016). Only a few of those studies have considered how hypoxia affects the aquatic larva during this morphological, behavioral, and physiological transition. What makes larval air breathing fishes particularly interesting in the study of hypoxia‐induced developmental plasticity is that at some point in their development such fishes have the ability to at least partially “escape” hypoxia and its consequences through the onset of air breathing. Whether this option of air breathing in later larval life makes early larvae prior to the onset of air breathing less or more developmentally plastic than strictly water breathing larval fishes is unknown.

The aim of this study, then, was to evaluate the effect of chronic hypoxia on larvae of the air‐breathing fishes the gourami (*Trichopodus trichopterus*) and the Siamese fighting fish (*Betta splendens)* prior to the onset of air breathing. *Trichopodus* and *Betta* are relatively closely related anabantids from the family Osphronemidae (Froese and Pauly 2016), both possessing a suprabranchial labyrinth organ that serves as the site of aerial respiration as juveniles and adults. Although both species use labyrinth organs for air breathing, these two air breathing fishes live and reproduce in distinctly different habitats. *Betta* breeds in temporal isolated ponds with standing waters located in flood plains such as rice paddies. These ponds are frequently hypoxic, even anoxic on the bottom, because of the high temperatures and high organic content (Froese and Pauly 2016; Monvises et al. 2009; Rainboth 1996). *Betta* is a bubble nest builder and males provide intense care for eggs and early larva (Monvises et al. 2009; Ruber et al. 2006). Young larval *Betta* are unable to escape aquatic hypoxia and may have a greater tolerance to aquatic hypoxia. In contrast to *Betta*,* Trichopodus* reproduces in lowland wetlands like marshes, swamps, and canals with seasonal floods that facilitate temporary lateral migrations from river mainstreams to seasonally flooded areas, with a return to the permanent water bodies as the dry season approaches (Froese and Pauly 2016; Rainboth 1996). Compared to *Betta*,* Trichopodus* larvae, and juveniles are more active swimmers based on our observations of larvae in holding tanks, potentially enabling migration back to rivers and other larger bodies of water from the increasingly hypoxic receding floodwater ponds.

Against this backdrop, we hypothesized that chronic environmental hypoxia will alter heart and opercular rate as well as $\dot{M}O_{2}$ and *P*_Crit_ in the larvae of both species, as in many strictly aquatic larval fishes. Additionally, we hypothesized that these physiological responses would differ qualitatively and/or quantitatively between *Trichopodus* and *Betta*, based on the different habitats of these two species, based in part upon their differential adjustments in larval onset of air breathing in response to hypoxia (Mendez‐Sanchez and Burggren 2014). To our knowledge this is the first study to consider development of larval air‐breathing fishes reared in the more natural condition of nocturnal hypoxia and diurnal normoxia.

## Material and Methods

### Rearing and maintenance

Large numbers of eggs are produced at each breeding in both *Betta* (500 eggs) and *Trichopodus* (1000–2000 eggs). Eggs hatch within 24–48 h and become free‐swimming in 3–4 days at 27°C (Mendez‐Sanchez and Burggren 2014; Pollak et al. 1981). Larval *Trichopodus* and *Betta* were maintained from hatching to 35 days postfertilization (dpf). Details of rearing protocol, water quality, and larvae maintenance have been previously described in detail (Mendez‐Sanchez and Burggren 2014).

All larvae for a single experiment were taken from the same clutch to avoid intraclutch effects. Day of fertilization was designated 0 dpf. For each subsequent 24 h cycle a unit of 1 day was added. Larvae were reared from hatching for 48 h in normoxia. Thereafter, larvae were transferred into different floating containers (250 mL) in 40 L aquaria and raised in either continuous normoxia (20 kPa) or in intermittent nocturnal hypoxia (17 and 14 kPa) until 35 dpf (*Trichopodus*) or 38 dpf (*Betta*). Oxygen levels of the water in the aquaria and in the containers containing the larvae were regulated by directing into the water a stream of either room air (control, 20 kPa) or a mixture of room air and nitrogen gas, creating hypoxia at levels of either 17 kPa or 14 kPa. Gas flows were regulated with flowmeters set to deliver the appropriate gas mixture.

The containers were sealed with Plexiglass covers with exhaust valves preventing atmospheric air from leaking into the containers. Each rearing container was filled with water to 80% of its capacity, with the remaining 20% receiving the gas emerging up from the water in the container. This configuration ensured that the gas and water phases of the containers were in PO_2_ equilibrium. Water PO_2_ was monitored daily using an optical oximeter probe ProODO (YSI Incorporated). PO_2_ of the gas phase was measured and monitored daily with a ProOx 110 oxygen sensor (Biospherix, Ltd).

Previous experiments with *Betta* and *Trichopodus* have revealed that even relatively mild hypoxia when delivered continuously produces very high larval mortality (Mendez‐Sanchez and Burggren 2014). Thus, the current experiments employed a far more natural regime of intermittent nocturnal hypoxia exposure synchronized with the experimental light:dark cycle. This protocol mimics the dial cycle of hypoxia in the tropical habitats were these fishes evolved. Specifically, all larval populations of both species were exposed to 12 h of normoxia (PO_2_ = 20 kPa) during the day. The control population also experienced only normoxia at night. Those designated as the mild hypoxia (17 kPa) or more severe hypoxia (14 kPa) populations were additionally exposed to their specified level of hypoxia for 12 h during the night. Each of the three PO_2_ populations was created in triplicate, with 150 larvae placed in three 0.25 L containers, each containing 50 larvae. Larvae for a particular physiological experiment were randomly sampled from one of the three containers. Once measurements were made, they were subsequently euthanized by submersion in a diluted solution of buffered MS222 (250 mg/L) until opercular movements stopped.

Larvae of the three populations were sampled for physiological variables at 5 day intervals until 35 dpf. Each larva was fasted for 12 h prior to any measurement. Because of the smaller clutch size of *Betta*, samples of this species were only taken from 20 to 35 dpf to ensure sufficient individuals for each $\dot{M}O_{2}$ measurement. The body mass and length of the larvae of both species are shown in Table 1.

**Table 1:** Mean ± standard error. *N* = 5 for each mean. The values correspond to the larvae used in the experimental measurements

Species	DPF	REARING PO_2_ (kPa)					
		14		17		20	
		Wet mass average (mg)	Length average (mm)	Wet mass average (mg)	Length average (mm)	Wet mass average (mg)	Length average (mm)
*Betta splendens*	20	1.80 ± 0.24	5.88 ± 0.74	1.90 ± 0.24	5.47 ± 0.74	2.27 ± 0.24	5.62 ± 0.74
	25	2.50 ± 0.24	6.31 ± 0.74	5.10 ± 0.21	6.96 ± 0.64	1.10 ± 0.24	5.06 ± 0.74
	30	2.90 ± 0.24	6.80 ± 0.74	6.13 ± 0.24	7.53 ± 0.74	5.40 ± 0.24	6.79 ± 0.74
	35	3.60 ± 0.24	6.98 ± 0.74	15.20 ± 0.24	10.49 ± 0.74	5.50 ± 0.24	7.31 ± 0.74
	38	7.09 ± 0.14	8.26 ± 0.43	7.80 ± 0.13	8.62 ± 0.41	10.49 ± 0.14	8.94 ± 0.43
*Trichopodus trichopterus*	5	0.26 ± 0.08	3.61 ± 0.42	0.26 ± 0.08	3.61 ± 0.42	0.26 ± 0.08	3.61 ± 0.42
	10	0.40 ± 0.12	3.37 ± 0.67	0.27 ± 0.10	3.55 ± 0.55	0.23 ± 0.10	3.21 ± 0.55
	15	0.57 ± 0.10	4.29 ± 0.55	0.50 ± 0.10	3.81 ± 0.55	0.50 ± 0.10	4.36 ± 0.55
	20	1.13 ± 0.10	5.89 ± 0.55	0.80 ± 0.10	5.41 ± 0.55	1.00 ± 0.10	5.52 ± 0.55
	25	0.93 ± 0.10	5.38 ± 0.55	0.97 ± 0.10	5.20 ± 0.55	1.33 ± 0.10	6.07 ± 0.55
	30	1.67 ± 0.10	6.02 ± 0.55	1.43 ± 0.10	5.94 ± 0.55	2.70 ± 0.10	6.93 ± 0.55
	35	5.11 ± 0.06	7.95 ± 0.33	3.35 ± 0.07	7.46 ± 0.39	3.61 ± 0.06	7.88 ± 0.33
	37	6.05 ± 0.07	8.14 ± 0.39	3.95 ± 0.05	7.73 ± 0.30	4.36 ± 0.05	7.71 ± 0.30

### Opercular rate and heart rate

Opercular rate (*f*_Op,_ opercular beats min^−1^) and heart rate (*f*_*H*_ *,* beats min^−1^) were measured at 27°C every 5 days from 5 to 35 dpf. In larvae of both *Betta* and *Trichogaster* the heart could be directly observed through the transparent body wall at these early ages. Larvae were gently placed in a 4.5 mL transparent flow‐through chamber for observation of opercular and heart rates. The chamber had water pumped at a rate of ~mL/min from the experimental rearing aquarium at the corresponding PO_2._ Pilot studies revealed that larvae returned to resting values of heart rate and gill ventilation with 10–15 min of gentle handling. Nonetheless, larvae were allowed to acclimate to the chambers for 1 h before measurements were begun. *f*_Op_ and *f*_*H*_ was recorded for 60 sec using a digital microscope (Celestron 44302‐A) at a magnification of 150x.

A 10 sec section of each video was analyzed with Tracker 4.72, an open source physics video analyzer (Brown, 2017). This software was used to automatically and simultaneously track heartbeat and operculum movements using changes in luminance (brightness in an image = the “black‐and‐white” or achromatic portion of the image) occurring through time in a selected area of the video. An example of the traces of *f*_Op_ and *f*_*H*_ obtained using Tracker 4.72 is shown in figure.

### Oxygen consumption

Every 5 days following fertilization, larvae from each treatment group were assessed for routine mass‐specific O_2_ consumption ($\dot{M}O_{2}$, *μ*mol·O_2_/g per hour) in normoxia (PO_2_ = 20 kPa) at 28°C, using intermittent closed respirometry via a Loligo Systems respirometry system (Tjele, Denmark) – see also (Lefevre et al. 2016). All $\dot{M}O_{2}$ measurements were made on 12 h fasting larvae during daylight hours in respirometers initially filled with normoxic water (PO_2_ = 20 kPa). Larvae were placed in a 2.0 mL borosilicate glass microrespirometer chamber and allowed to acclimate for 1 h in the respirometer while water was gently refreshed from the outer reservoir using a peristaltic pump. Four chambers were used simultaneously. One chamber for each contained only aerated water and served as blank sample to determine the effects of possible microbial respiration. The designated blank chamber was rotated through all four chambers during the course of four runs. Inevitably, microbial oxygen consumption was below the system's detection level (accurate to<<0.0005 *μ*mol·O_2_/g per hour). For determination of routine $\dot{M}O_{2}$, larvae were placed in aerated water, and the decline in PO_2_ (typically 1–3 kPa) in each respirometer containing a fish and the blank was measured for 2–6 h, depending on the larva's O_2_ consumption. PO_2_ decline was measured using temperature‐compensated fiber‐optic planar sensors attached to an OXY‐4 meter (PreSens). The signals from these probes were displayed on a computer with the automated data acquisition system DAQ‐M. The AutoResp software (Loligo Systems ApS) calculated mass‐specific oxygen consumption from the rate of O_2_ decline in each chamber over time, the elapsed time, the chamber volume, and the fish mass. This $\dot{M}O_{2}$ was designated routine metabolic rate (RMR) since although there was little or no effect of specific dynamic action associated with feeding given the fasting nature of the fish, the activity level of the fishes could not be directly observed in the individual respirometers. However, separate observations of larval *Betta* indicated that locomotor movement was infrequent for the first 35 dpf, suggest that our measured RMR was close to standard metabolic rate, at least for this species.

Critical oxygen tension (*P*_Crit_, in kPa) is the oxygen partial pressure (PO_2_) at which routine $\dot{M}O_{2}$ can no longer be maintained at normoxic levels as PO_2_ further decreases. *P*_Crit_ was measured using the same experimental design and apparatus as for measurement of routine $\dot{M}O_{2}$ but using closed respirometry as opposed to intermittent flow‐through respirometry. PO_2_ in the respirometers was allowed to fall to close to 0 kPa. A two‐phase linear regression model (Mueller et al. 2011; Yeager and Ulstch 1989) was applied to the PO_2_ versus $\dot{M}O_{2}$ data. To avoid the confounding effects of intraclutch variation, *P*_Crit_ was only measured in six groups originally obtained from the same egg clutch. *P*_Crit_ was determined on the first day at which air breathing was observed (~35 pdf).

### Statistical analysis

MANOVA comparisons were performed on $\dot{M}O_{2}$ *P*_Crit_ *, f*_*H*_, and *f*_Op_ using PO_2_, age and, in some cases, species, as factors. MANCOVA was also performed on $\dot{M}O_{2}$ to correct for the effect of age, using days of postfertilization as a covariate. To compare the effect of PO_2_ on the relationship between *f*_*H*_ and *f*_Op_, linear comparison of slopes and intersections were also utilized.

Treatment groups were considered significantly different at *P *<* *0.05. All data are expressed as mean ± 1 standard error (SE). N values are indicated for individual means, and total numbers of larvae used are indicated in the statistical descriptions.

All larval fishes used in this research were treated according to U.S. federal regulations and guidelines. This study was approved and monitored by the University of North Texas Animal Care and Use Committee (Project number 1111‐16).

## Results

### Opercular ventilation rate

**Figure 1:**
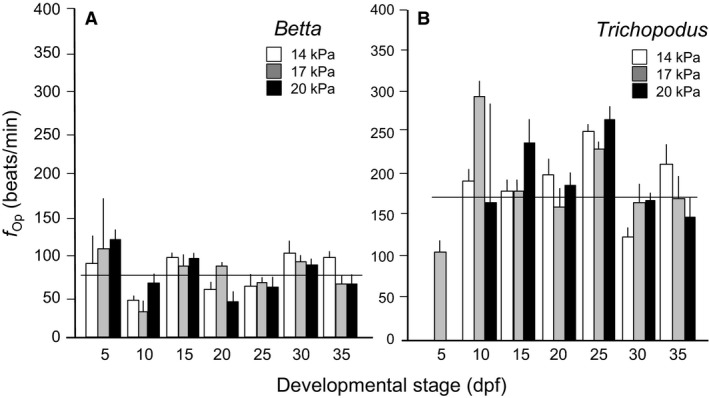
Comparison of opercular rate (*f*_O*p*_) from 5 days through 35 days postfertilization in (A) *Betta splendens* and (B) *Trichopodus trichogaster* reared in three different levels of PO_2_. Means ± SE are presented. *N* = 9. The horizontal lines represent the average *f*_*H*_ for all ages.

1. *Normoxic development*: Larval *Betta* reared and measured in normoxia were rapidly and extensively ventilating their gills at 5 dpf, the first day of measurement, and had completely consumed their egg yolk. *f*_Op_ in larval *Betta* did not significantly vary through development (*F* = 0.4, df = 2, 63, and *P *>* *0.05), averaging 77 ± 3 opercular beats/min over the developmental span monitored (Fig. 1A). In contrast to *Betta*, larval *Trichopodus* reared and measured in normoxia had not yet established opercular beating on dpf 5 (Fig. 1B). However, once opercular movements began much later at 10 dpf, the overall *f*_Op_ average value of 173 ± 6 beats/min was higher in *Trichopodus* than in *Betta* at any given developmental stage.
2. *Chronic hypoxia*: Rearing in chronic hypoxia (combined aquatic and aerial) did not induce any significant changes in gill ventilation measured in normoxia in larval *Betta* (*F* = 7, *df* = 6, 63, and *P *>* *0.05) (Fig. 1A). Although in the normoxic population of *Trichopodus* there was no opercular beating at 5 dpf, the dpf 5 *Trichopodus* chronically exposed to mild hypoxia (PO_2 _= 17 kPa of) were actually exhibiting opercular beating (Fig. 1B). Surprisingly, however, the population reared in an even lower level of oxygen (14 kPa) was not yet ventilating their gills at dpf 5. From 10 dpf onwards all three populations of *Trichopodus* were actively ventilating their gills, but there were no significant variation associated with level of hypoxia (*F* = 0.6, df = 2, 60, and *P *>* *0.05) (Fig. 1B).

### Heart rate

**Figure 2:**
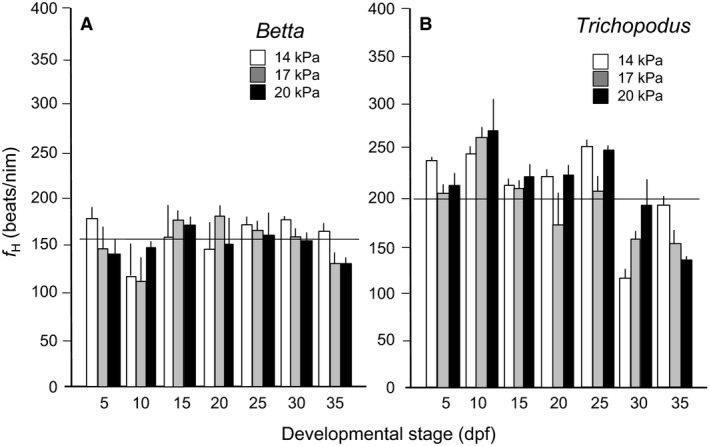
A comparison of heart rate (*f*_H_) in larval *Betta splendens* (A) and *Trichopodus trichogaster* (B) through 35 days postfertilization in three levels of PO_2_. Means ± SE are presented. *N* = 9. The horizontal lines represent the average *f*_*H*_ for all ages.

1. *Normoxic development*: Heart rate in larval *Betta* reared and measured in normoxia showed no significant changes as a function of development (*F* = 0.4, *df* = 2, 63, and *P *>* *0.05) (Fig. 2A), with overall heart rate averaging 156 ± 4 beats/min. This value was ˜25% lower than for *Trichopodus* at all comparable stages of development, where overall *f*_*H*_ across the entire monitored period was 212 ± 3 beats/min. Unlike for *Betta*, however, *f*_*H*_ in *Trichopodus* decreased slowly and significantly with development (*F* = 29.1, df = 2, 60, and *P *<* *0.0001) (Fig. 2B).
2. *Hypoxic development*: Heart rate in larvae of both *Betta* and Trichopoduschronically reared in hypoxia showed no significant changes as a function of rearing oxygen level (*F* = 0.4, *df* = 2, 63, and *P *>* *0.05) and (*F* = 0.6, *df* = 2, 60, and *P *>* *0.05) (Fig. 2).

### Heart beat‐opercular rate relationship

**Figure 3:**
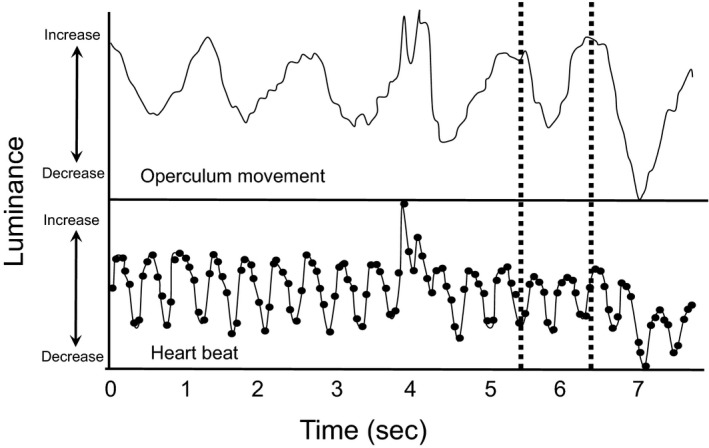
Representative traces taken from software analysis of video images of *Betta splendens* (35 days postfertilization) raised in hypoxia (17 kPa). The dashed vertical lines toward the right of the panels illustrate the ~2:1 ratio of heart beat to opercular movement in this trace. See text for additional details.

**Figure 4:**
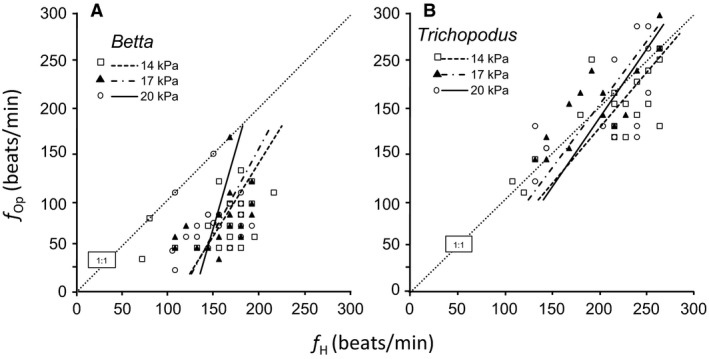
The heart rate:opercular rate relationship (*f*_*H*_: *f*_Op_) in larvae of (A) *Betta splendens* and (B) *Trichopodus trichopterus*. Data from 5 dpf to 35 dpf were pooled, since preliminary analysis revealed no significant effects of development. The lines show the data for three groups reared in different levels of PO_2_. *n* = 20 for each PO_2_ group of each species. In all cases *r* > 0.9 and *P *<* *0.05 indicating significance of relationship. See text for statistical analysis of differences between experimental groups.

Interactions between heart rate and opercular rate are often taken as an indication of neural cardiorespiratory coordination in fishes (Dick et al. 2014; Schulz et al. 2013; Taylor 1985; Taylor et al. 2006). Representative traces of simultaneously recorded opercular rate and heart rate in a dpf 35 *Betta splendens* are shown in Figure 3. In part because of considerable variations in duration of the opercular cycle within individual fish, the interaction of *f*_*H*_ and *f*_*Op*_ showed no differences across ages in either *Betta* or *Trichopodus*, with the sole exception of dpf 5 *Trichopodus*. Consequently, the effect of this factor was not considered from the perspective of their interaction, and data for the *f*_*H*_ *:f*_*Op*_ relationship was determined from a pool of all individuals from 5 to 35 dpf. From this general population it was determined that the interaction of *f*_*H*_ and *f*_Op_ was significantly affected by hypoxia rearing level in both *Betta* and *Trichopodus*. Larval *Betta* reared in normoxia varied significantly from the line of identity (1:1 ratio), showing an approximate timing of 3 heartbeats per 1 opercular beat (Fig. 4A). However, rearing in either level of chronic hypoxia resulted in patterns of *f*_*H*_:*f*_Op_ ratio of ~2:1, which were significantly different from each other, from the 1:1 ratio, and from the line describing the normoxic ratio (*F*_slopes _= 2.57; df = 3,7; *P *<* *0.05 and *F*_intercepts _= 14.02; df = 3,7; *P *<* *0.001). Essentially, hypoxic treatments increased *f*_*Op*_ compared with the normoxic larva with the same *f*_*H*_. Larval *Betta* also tended to have a *f*_*H*_:*f*_Op_ with a slope more similar to the 1:1 ratio than to the normoxic slope. For larval *Trichopodus* the *f*_*H*_:*f*_Op_ relationship was statistically identical to a 1:1 ratio (Fig. 4B), and showed no significant differences between larvae reared in normoxia and either level of hypoxia larvae (*F*_slopes _= 1.62; df = 3,7; *P *>* *0.05 and *F*_intercepts _= 0.83; df = 3,7; *P *>* *0.05).

### Mass‐specific O_2_ consumption

**Figure 5:**
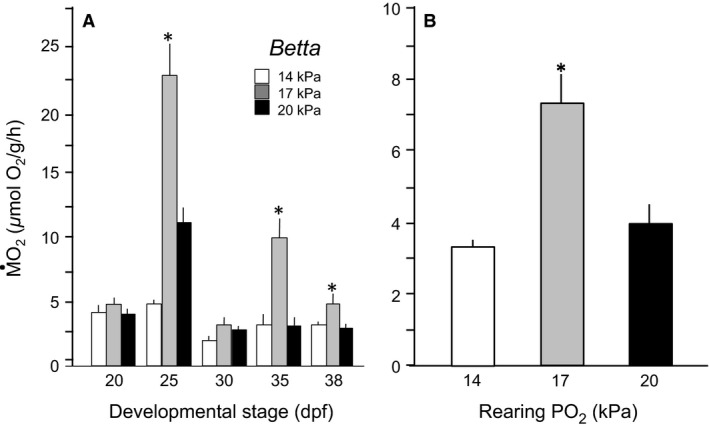
Routine oxygen consumption ($\dot{M}O_{2}$) of larval *Betta splendens*. (A) $\dot{M}O_{2}$from 20 through 20–35 dpf reared in three levels of PO_2_.(B) $\dot{M}O_{2}$ corrected for aged differences in the three larval populations. Means ± SE are presented. *n* = 9. An * indicates a significant difference from control (20 kPa).

**Figure 6:**
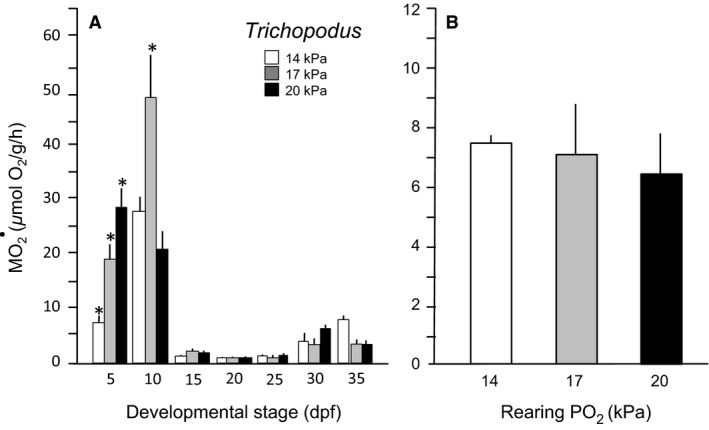
Routine oxygen consumption of $\dot{M}O_{2}$ larval *Trichopodus trichopterus*. (A) $\dot{M}O_{2}$through 35 dpf reared in three levels of *P*O_2_. The horizontal line indicates the average value across all developmental stages. (B) $\dot{M}O_{2}$ corrected for aged differences in the three larval populations. Means ± SE are presented. *n* = 9. An * indicates a significant difference from control.

1. *Normoxic development*: Larval *Betta* showed a complex pattern of developmental change in routine oxygen consumption ($\dot{M}O_{2}$) under normoxic conditions, with significantly higher values at early stages and declining values later in larval development (*F* = 74, df = 4, 228, and *P *<* *0.0001) (Fig. 5A). *Trichopodus* similarly presented higher routine $\dot{M}O_{2}s$ during earliest stages of development (*F* = 137, df = 6, 167, and *P *<* *0.0001) (Fig. 6A), but quickly settled into a relatively stable routine $\dot{M}O_{2}$ ˜9.03 *μ*mol O_2_/g per hour throughout the rest of development (Fig. 6A). This level of $\dot{M}O_{2}$ was approximately 60% higher than in *Betta* over comparable developmental stages.
2. Hypoxic development. The effects of hypoxic rearing were complex in *Betta*, as were the changes during its normoxic development. Mild chronic hypoxic exposure (17 kPa) actually stimulated routine $\dot{M}O_{2}$ above control (normoxic) levels. However, more severe chronic hypoxic rearing (14 kPa) in *Betta* depressed routine $\dot{M}O_{2}$ at all developmental stages (*F* = 73.9, df = 2, 228, and *P *<* *0.0001) (Fig. 5A). Analyzing the effect of hypoxia on $\dot{M}O_{2}$ corrected for age (i.e., using age as a covariate) in *Betta* revealed a significant effect of hypoxia in the mildly hypoxic 17 kPa group (*F* = 18.2, df = 2, 227, and *P *<* *0.0001) (Fig. 5B). In stark contrast to *Betta*, however, in *Trichopodus* neither level of chronic hypoxia induced significant changes in $\dot{M}O_{2}$, confirmed by analyzing the effect of hypoxia corrected for age (*F* = 0.2, df = 2, 167, and *P *>* *0.05) (Fig. 6B).

### Critical oxygen partial pressure (*P*_Crit_)

**Figure 7:**
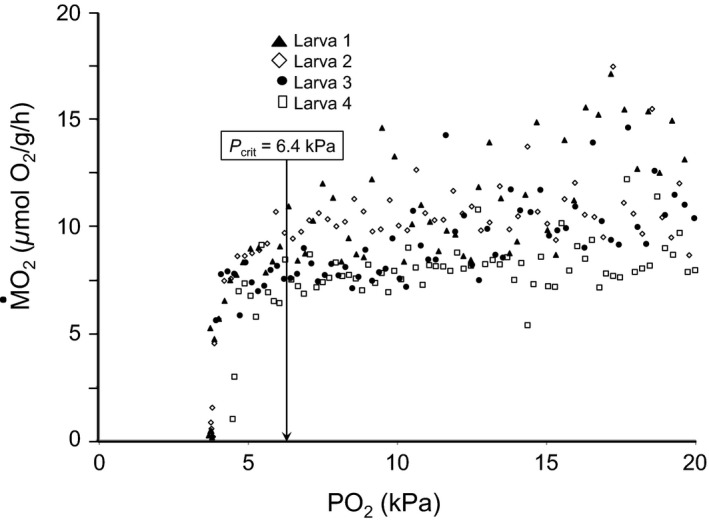
Representative traces of routine $\dot{M}O_{2}$ with declining PO_2_ in four larval *Trichopodus* (35 dpf) reared in chronic intermittent hypoxia (14 kPa). Different symbols represents a different larva, and each individual symbol represents a single measurement of $\dot{M}O_{2}$ at the indicated PO_2_.

**Figure 8:**
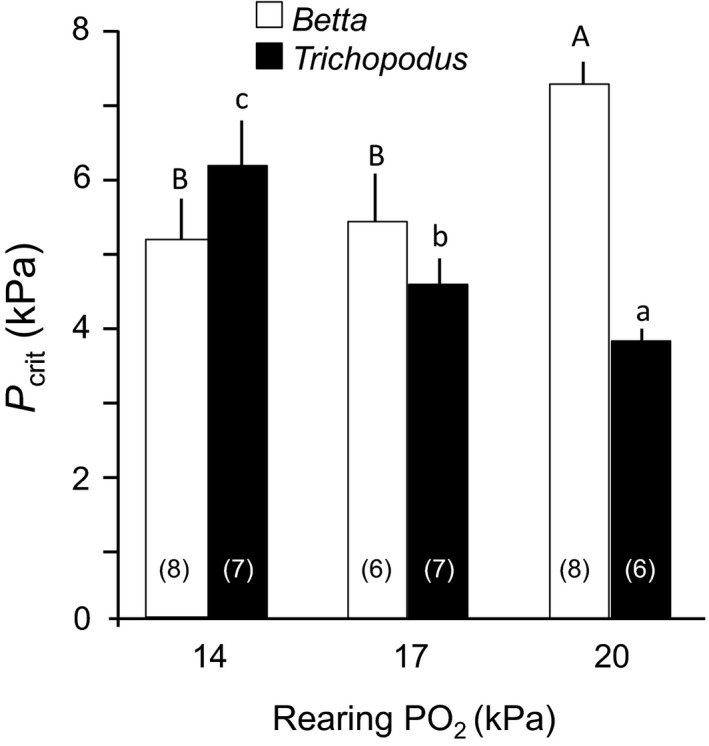
Comparison of *P*_Crit_ for larval *Betta* and *Trichopodus* reared in different levels of PO_2_ at 35 dpf. Boxes enclose statistically identical means (*P *>* *0.05). n values for each group are in parentheses.

An example of the raw data for the calculation of *P*_Crit_ is shown in Figure 7. *Betta* and *Trichopodus* showed two different, opposing patterns for the effects of rearing oxygen level on *P*_Crit_ measured on dpf 35 (Fig. 8). In larval *Betta*,* P*_Crit_ was positively correlated with rearing PO_2_ (*F* = 5.5, df = 2, 21, and *P *<* *0.05), with *P*_Crit_ at 14 kPa 37% lower than the value in the normoxic population. In contrast, in *Trichopodus* larvae the *P*_Crit_ actually increased as rearing conditions grew more hypoxic (*F* = 17, df = 2, 19, and *P *<* *0.0001), with chronic rearing at a PO_2_ of 17 and 14 kPa PO_2_ increasing *P*_Crit_ to higher PO_2_s by 24% and 70%, respectively.

## Discussion

### Development in Normoxia

The ventilatory frequency (*f*_Op_) of larval *Betta* and *Trichopodus* in normoxia was ~80 and ~175 beats/min, respectively. Although these two values cover a fairly wide range of opercular rates, they fall within the wide range of values for larvae of other aquatic freshwater teleost fishes at similar temperatures or when corrected for different temperatures using a Q10 of 2 – e.g., (Burggren et al. 2016; Holeton 1971; Lerner et al. 2007; Vosyliene et al. 2005). These gill ventilation rates of larvae are considerably higher than ventilation rates in the much larger adults of the same species of either strictly aquatic or air breathing fishes – for example, (Cerezo et al. 2006; Kalinin et al. 2000; McKenzie et al. 2007; Porteus et al. 2011; Richards and Haswell 2011), as would be expected from scaling effects on physiological processes.

Heart rate in larval *Betta* and *Trichopodus* was ~160 and ~210 beats/min, respectively. Relatively few measurements of heart rate exist for the larvae of either air breathing or strictly aquatic species, but the rates recorded in this study are within the range of those recorded at similar temperatures (27–28̊°C) in another larval air‐breathing fish, the tropical gar *Atractosteus tropicus* (Burggren et al. 2016) and in unrestrained, larval zebrafish (*Danio rerio*), for example, (Barrionuevo and Burggren 1999; Jacob et al. 2002; Kopp et al. 2014; Miller et al. 2014; Parker et al. 2014; Rider et al. 2012; Rombough 2007; Steele et al. 2011; Velasco‐Santamaria et al. 2011). In this respect, the earliest strictly aquatic stages of these air‐breathing fishes physiologically resemble those of aquatic freshwater fishes, generally.

Both opercular rate and heart rates typically change over early development in vertebrates, showing complex patterns of change that are often characterized by an initial increase during the early phases of organogenesis followed by declines predicted from allometric scaling (Burggren and Warburton 2005; Burggren 2005). Yet, in this study neither opercular nor heart rates showed any major changes during development in either *Betta* or *Trichopodus*. This may suggest a lack of neural and hormonal control of both parameters during the first 35 days of postfertilization (McKenzie et al. 2007; Taylor et al. 2010) prior to the onset of air breathing in these species (Mendez‐Sanchez and Burggren 2014). This would be a much later development of respiratory reflexes than in the air‐breathing tropical gar, *Atractosteus tropicus*, but this species starts air breathing far earlier at around 4–5 dpf at similar temperatures (Burggren et al. 2016). In any event, challenge by stressors (e.g., temperature, oxygen, activity) will be required to confirm the specific timing of onset of cardiorespiratory regulation in *Betta* and *Trichopodus*.

Coupling of cardiac and respiratory activity has long been appreciated, and gives insights into the maturity and complexity of physiological regulatory systems (Dick et al. 2014; Schulz et al. 2013; Taylor 1985; Taylor et al. 2006). The *f*_*H*_:*f*_Op_ relationship for larval *Betta* was approximately 3 heartbeats for every 1 opercular beat. The *f*_*H*_:*f*_Op_ ratio in larval *Trichopodus* was much lower at ~1:1. It has been speculated that a coordination between ventilation and heart rate in normoxia may improve gas transport across the gills by matching convective delivery of oxygen to the gills with the ability of the perfusing blood to remove it (Rombough 1998; Smatresk et al. 1986). This would potentially making *Trichopodus* more efficient in extracting O_2_ from the water. In the adults of the facultative air‐breathing fish, *Lepisosteus osseus* (Rahn et al. 1971), the coupling between heart rate and gill ventilation ratio in normoxia was also ~1:1 (Smatresk et al. 1986). For adults of another facultative air‐breather *Hoplerythrinus unitaeniatus* (Oliveira et al. 2004), the mean ventilatory frequency was slightly more than twice the heart frequency, a typical relation for a strictly water breathing fish (McKenzie et al. 2007). For the hypoxia tolerant adult water breather *Piaractus mesopotamicus*,* f*_*H*_:*f*_Op_ approximated 3:1 (Leite et al. 2007; Taylor et al. 2009). Clearly, larval *Betta* showed less opercular movements per heart beat than the other two species of facultative air breathers employing aquatic respiration. This could be related to the low level of physical activity we have observed in larval *Betta*. Thus, in normoxic conditions the high *f*_*H:*_ *f*_Op_ ratio could allow adequate tissue O_2_ delivery. Also, during early developmental stages skin breathing is likely also directly involved in providing gas exchange (see (Blank and Burggren 2014; Feder and Burggren 1985; Liem 1981; Wells and Pinder 1996b), deemphasizing cardiorespiratory coupling in early developmental stages. However, a definitive answer on the importance cardiorespiratory coupling in developing, as opposed to adult, fishes will have to await additional data.

Larval *Trichopodus* had a routine of $\dot{M}O_{2}$~9.03 *μ*mol O_2_/g per hour, which was considerably higher than larval *Betta,* 4.8 ± 0.34 *μ*mol O_2_/g per hour. In fact, the routine $\dot{M}O_{2}$ of larval gourami is in the range of the larvae of active strictly aquatic fishes. The mean routine $\dot{M}O_{2}$ of larval *Betta* was between reported values of larval air breathers and water breathers (Bagatto et al. 2001; Barrionuevo et al. 2010; Gore and Burggren 2012; Graham 1997; Lucas et al. 2014; Peters 1978; Rombough 1998; Wells and Pinder 1996a). Simple observation of larval *Trichopodus* raised in normoxia reveals them to be active swimmers with a high oxygen demand, compared with larval *Betta* which are much more likely to rest on the substrate (Mendez‐Sanchez and Burggren 2014),

*Trichopodus* and *Betta* occupy considerably different positions on the behavioral and locomotor gradient of fish lifestyles (Dwyer et al. 2014; Stoffels 2015). The slower lifestyle of *Betta*, which is an ambush predator or “saltatory” forager, has mostly benthic habits, yet is capable of rapid starts and turns for high‐acceleration prey capture. Consequently, low metabolic rates and high P_Crit_ values characterize the metabolism of *Betta*.

The *P*_Crit_ of larval *Betta* was 7.2 ± 0.4 kPa. This compares with 4.6–8.1 kPa for larval *Hoplosternum littorale* (Sloman et al. 2009), 9.3 ± 1.0 kPa for adults of the facultative air‐breathing *Amia calva* (Porteus et al. 2014), and 2–6 kPa for adults of *Mogurnda adspersa, Melanotaenia fluviatilis,* and *Hypseleotris sp*. exposed to natural hypoxia episodes as a consequence of droughts (Stoffels 2015). *P*_crit_ for larvae of the aquatic *Danio rerio* are in the range of 7.3–9.9 kPa (Barrionuevo and Burggren 1999; Barrionuevo et al. 2010). *P*_Crit_ has been considered to be an indicator of hypoxia tolerance (Chapman et al. 2002; Mandic et al. 2013). Again, this characteristic for *Betta* is in the middle of a range of aquatic and facultative air‐breathing fishes, pointing to its facultative air‐breathing habit. An intermediate *P*_Crit_ makes easier the possibility to adjust its respiratory performance to either a water or air‐breathing strategy (Robertson et al. 2014).

Once again in contrast to *Betta*,* Trichopodus* fits with a faster lifestyle – an active cruising and pursuit predator with endurance swimming and sprints for sustained chases, patrolling, drift feeding, searching, etc. These traits are reflected in this species higher metabolic rates and low P_Crit_, which are characteristic of more rapidly swimming fishes.

### Development in chronic intermittent hypoxia

Different fish species employ different approaches for dealing with aquatic hypoxia. Hypoxia resistance, the ability to actively maintain O_2_ extraction and thus routine metabolic rate even as O_2_ levels fall, allows animals to exploit environments with variable O_2_ levels (Mandic et al. 2013). The hyperventilation reflex is critical for this strategy. However, some species exhibit hypoxic tolerance (distinct from resistance), whereby as environmental PO_2_ falls, the fish no longer actively maintains normal rates of aerobic O_2_ consumption. This requires the ability to tolerate increasing levels of tissue hypoxia (Perry 2011), and is often correlated with lower critical PO_2_ (Chapman et al. 2002; Mandic et al. 2013). *Betta* and *Trichopodus* both responded physiologically to rearing in chronic intermittent hypoxia, but with highly species‐specific differences, as will now be considered.

### *Betta*

In *Betta* reared under mild hypoxia (PO_2 = _17 kPa), routine $\dot{M}O_{2}$ was elevated at most stages of development (Fig. 5), showing that these larvae can regulate and increase aquatic oxygen consumption, even at an early larval stage. In this respect, larval *Betta* appears to be like the larvae of active aquatic species such as *Danio rerio,* whose oxygen uptake appears to be enhanced by mild hypoxia (Barrionuevo and Burggren 1999; Barrionuevo et al. 2010). However, routine $\dot{M}O_{2}$ in *Betta* was reduced compared to normoxia under more severe oxygen stress (14 kPa) (Fig. 6). Physiological adjustments to oxygen extraction from hypoxic water to maintain homeostasis are expensive (Perry 2011; Perry et al. 2009) and the cost cannot easily be sustained in more severe hypoxia. In any event, the low routine $\dot{M}O_{2}$ presented by larval *Betta* at 14 kPa also reflects their limited ability to maintain O_2_ uptake at this level of hypoxia, which is reflected in low survival at this PO_2_ in both continuous and intermittent hypoxia (Mendez‐Sanchez and Burggren 2014).

Larval *Betta* chronically reared under hypoxic conditions showed a *P*_Crit_ that was 30% lower than when reared in normoxia. A lower *P*_Crit_reflects the ability of larval *Betta* to continue extracting O_2_ at progressively lower oxygen levels, essentially making them more resistant to hypoxia and correlating with that fact that this species has evolved in more hypoxic waters than *Trichopodus* (Froese and Pauly 2016; Monvises et al. 2009; Rainboth 1996).

Facultative air breathers evolved the air‐breathing habit in response to unpredictable environmental conditions, especially with respect to ambient temperature and oxygen (Brauner et al. 2004; Graham 1997; Randall et al. 1981). To be a facultative air breather implies the ability to adjust physiological variables in the face of unpredictable environment situations. Such ability was evident in larval *Betta* when examining the *f*_*H*_:*f*_Op_ relationship and how this variable was affected by hypoxic rearing. Chronic hypoxia induced higher levels of *f*_Op_ at the same *f*_*H*_, and the relationship between these two variables changed from 3:1 in normoxia to 2:1 in hypoxia. This relative increase in perfusion compared to ventilation may also improve gas transport across the gills (Smatresk et al. 1986), potentially making this species more efficient in extracting O_2_ from the water under hypoxic conditions.

### *Trichopodus*

Larval *Trichopodus*, unlike larval *Betta*, showed little to no physiological response to chronic aquatic hypoxia, a lack of physiological plasticity potentially making this species less tolerant to hypoxia (Chapman et al. 2002; Mandic et al. 2013; Perry 2011). This characteristic may also explain the low overall survival of larval *Trichopodus* in hypoxia (Mendez‐Sanchez and Burggren 2014). As an obligate air breather, the only mechanism that larval *Trichopodus* have to escape aquatic hypoxia is to resort to air‐breathing (Graham 1997). Rearing in chronic hypoxia had no significant effect on the routine $\dot{M}O_{2}$ of larval Trichopodusbut did increase *P*_Crit_. Thus, *Trichopodus* showed less hypoxia tolerance when reared in a more hypoxic environment. The inability of larval *Trichopodus* to maintain aquatic oxygen consumption is likely correlated with fact that the juvenile and adult *Trichopodus* are obligate air breathers (Graham 1997).

Collectively, then, the physiological responses to hypoxia exhibited by *Betta* can be considered more plastic than *Trichopodus*, allowing enhanced physiological compensation to environmental hypoxia (West‐Eberhard 2005). Larval *Betta* was able to adjust $\dot{M}O_{2}$, P_Crit_, *f*_*H*_, and *f*_*Op*_ in response to hypoxia to increase its ability to withstand hypoxia. Facultative air breathers might be expected to be more plastic in their responses, given that they have two options for environmentally derived oxygen – air or water – and to some extent with enhanced plasticity they could emphasize one respiratory approach over another depending upon environmental conditions (Graham 1997). This larval developmental phenotypic plasticity induced by hypoxia in *Betta* similarly occurs in the fully aquatic *Danio rerio*, in which its increased hypoxia tolerance (lower P_Crit_) was associated with the induction of HIF‐1 during critical developmental windows (Robertson et al. 2014).

### Respiratory developmental plasticity and physiological heterokairy

Interestingly, in larval *Trichopodus* (but not *Betta*) chronic rearing in mild hypoxia actually advanced the onset of opercular beating to before 5 dpf, compared to 10 dpf in the normoxic population. This response, apparently adaptive in that it would allow earlier access to aquatic oxygen, provides another example of heterokairy, along with the advancement of the onset of air breathing (Mendez‐Sanchez and Burggren 2014). Heterokairy, a form of developmental phenotypic plasticity within populations or individuals, is the change in the timing of the onset of development, especially of physiological regulatory systems and their components, at the individual or population level (Spicer and Burggren 2003; Spicer et al. 2011). Such examples have mostly been shown in invertebrates, so the observation of this phenomenon in a vertebrate is noteworthy.

In conclusion, these physiological data indicate that larval *Betta* is in many ways better adapted to aquatic survival than larval *Trichopodus*. This correlates well with the more hypoxic habitats typically inhabited by larval *Betta*.

## Conflict of Interest

The authors declare no conflicts of interest.
